# Supplementary figures and images for: East palearctic treefrog past and present habitat suitability using ecological niche models
Source: PeerJ. 2022 Mar 3;10:e12999. doi: 10.7717/peerj.12999 (PMC8898549; doi:10.7717/peerj.12999)

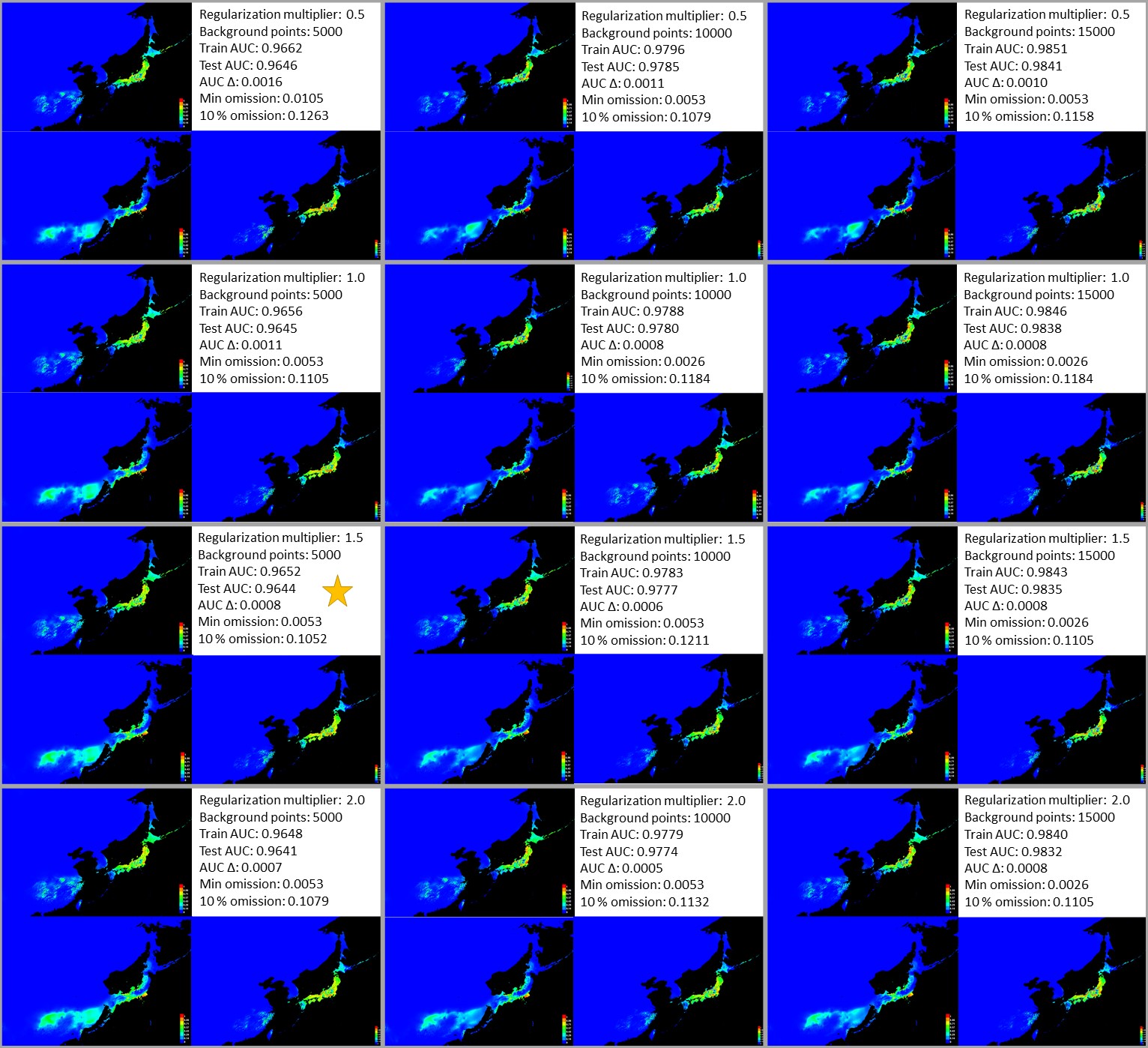

Supplement: Supplemental Information 1 — Metrics (top right of each panel) include regularization multiplier, number of background points, training AUC, test AUC, AUC∆, minimum test omission and 10% percent test omission. The selected model is marked by a gold star. [file peerj-10-12999-s001.jpg]

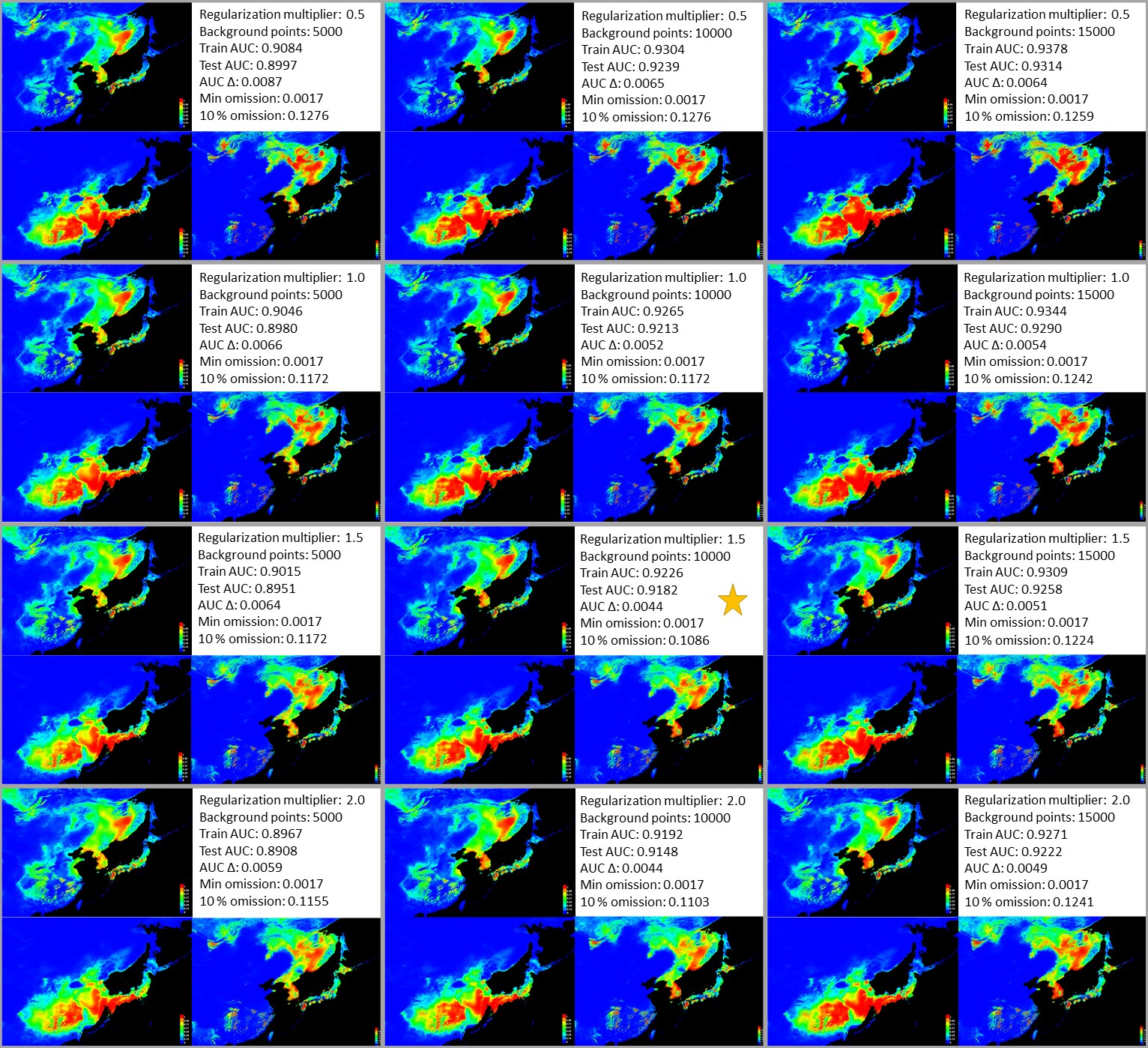

Supplement: Supplemental Information 2 — Metrics (top right of each panel) include regularization multiplier, number of background points, training AUC, test AUC, AUC∆, minimum test omission and 10% percent test omission. The selected model is marked by a gold star. [file peerj-10-12999-s002.jpg]

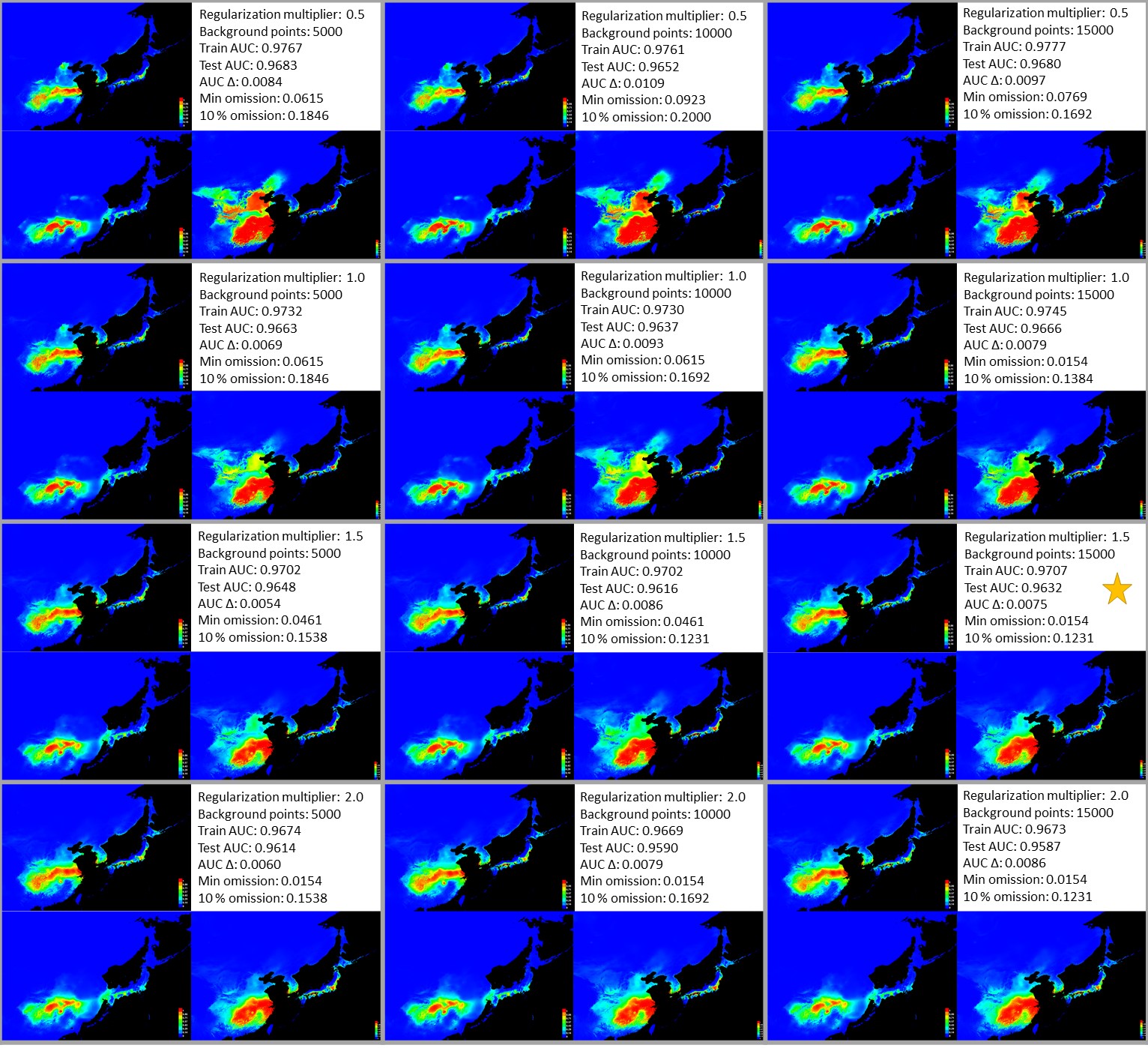

Supplement: Supplemental Information 3 — Metrics (top right of each panel) include regularization multiplier, number of background points, training AUC, test AUC, AUC∆, minimum test omission and 10% percent test omission. The selected model is marked by a gold star. [file peerj-10-12999-s003.jpg]

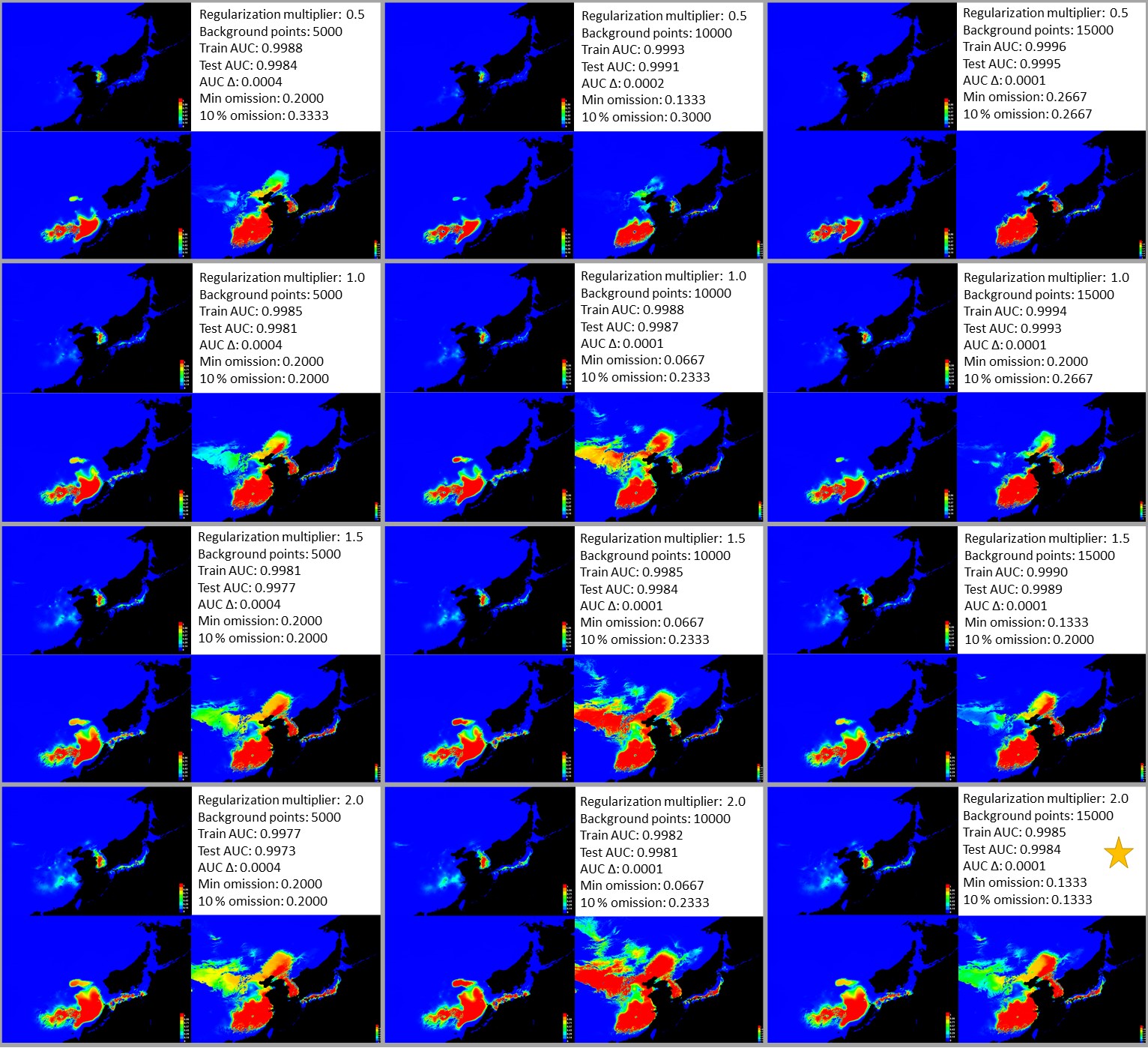

Supplement: Supplemental Information 4 — Metrics (top right of each panel) include regularization multiplier, number of background points, training AUC, test AUC, AUC∆, minimum test omission and 10% percent test omission. The selected model is marked by a gold star. [file peerj-10-12999-s004.jpg]

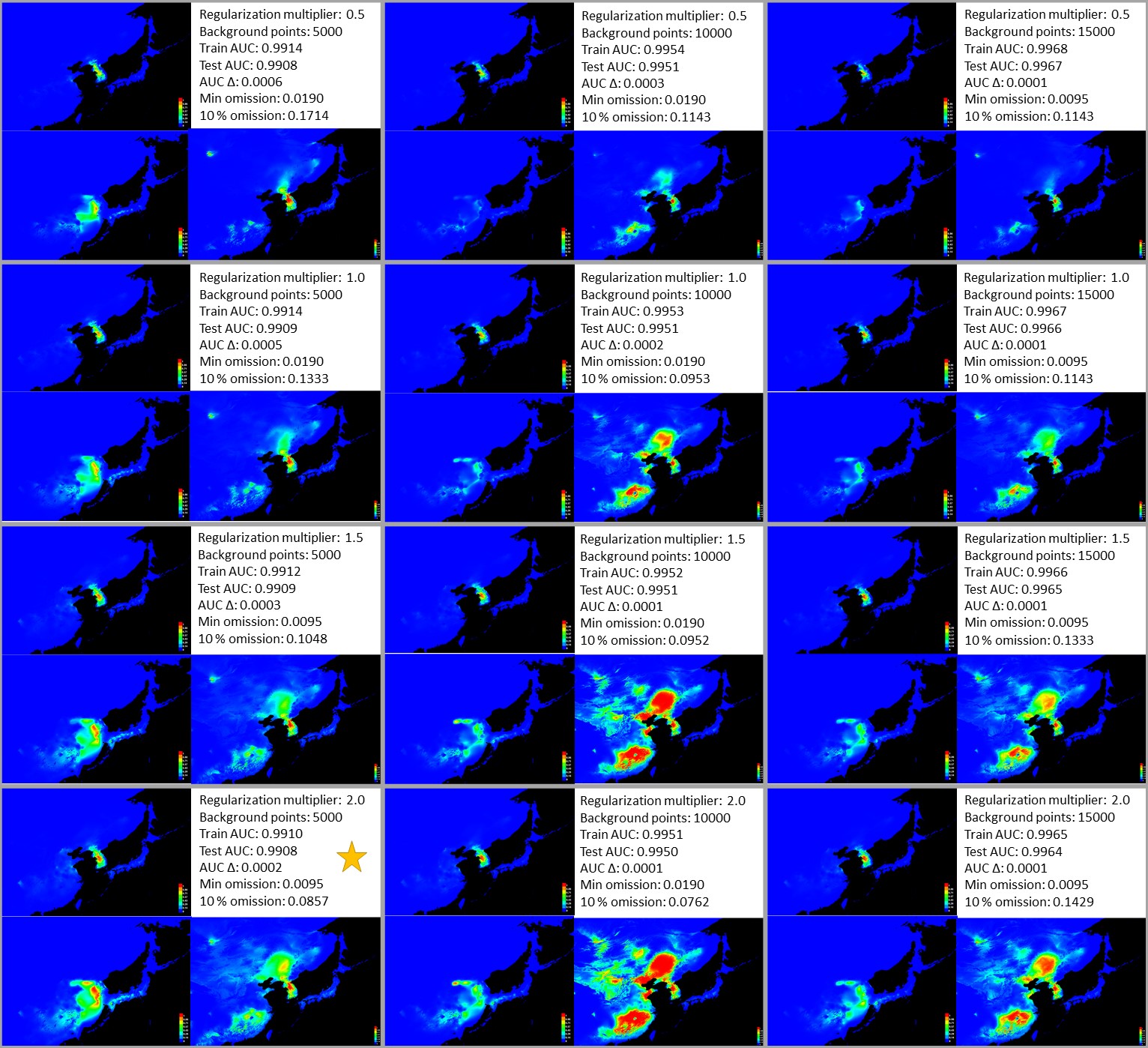

Supplement: Supplemental Information 5 — Metrics (top right of each panel) include regularization multiplier, number of background points, training AUC, test AUC, AUC∆, minimum test omission and 10% percent test omission. The selected model is marked by a gold star. [file peerj-10-12999-s005.jpg]

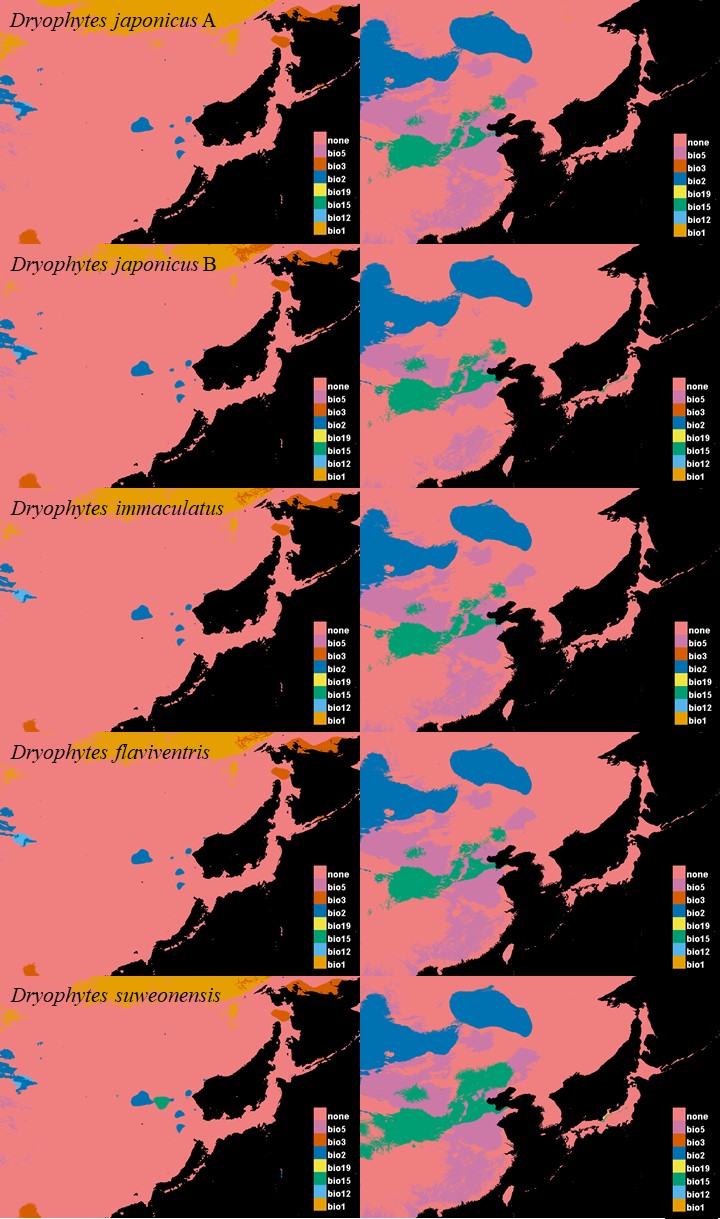

Supplement: Supplemental Information 6 [file peerj-10-12999-s006.jpg]
