# Supplementary material for: East palearctic treefrog past and present habitat suitability using ecological niche models
Source: PeerJ. 2022 Mar 3;10:e12999. doi: 10.7717/peerj.12999 (PMC8898549; doi:10.7717/peerj.12999)
Supplement: Supplemental Information 7 — Overfitting statistics (AUC ∆, minimum test omission and 10% test omission) for selected maximum entropy models for five east palearctic Dryophytes clades. [file peerj-10-12999-s007.docx]

| Clade | AUC ∆ | Minimum omission | 10% omission |
| --- | --- | --- | --- |
| *Dryophytes japonicus* (Clade A) | 0.0008 | 0.0053 | 0.1052 |
| *Dryophytes japonicus* (Clade B) | 0.0044 | 0.0017 | 0.1086 |
| *Dryophytes immaculatus* | 0.0075 | 0.0154 | 0.1231 |
| *Dryophytes flaviventris* | 0.0001 | 0.1333 | 0.1333 |
| *Dryophytes suweonensis* | 0.0002 | 0.0095 | 0.0857 |
